# Supplementary material for: Graded intrafillable architecture-based iontronic pressure sensor with ultra-broad-range high sensitivity
Source: Nat Commun. 2020 Jan 10;11:209. doi: 10.1038/s41467-019-14054-9 (PMC6954251; doi:10.1038/s41467-019-14054-9)
Supplement: Supplementary file 1 — Supplementary Information [file 41467_2019_14054_MOESM1_ESM.pdf]

# Supplementary Information for

**Graded Intrafillable Architecture-based Iontronic Pressure Sensor with Ultra-Broad-Range High Sensitivity**

Ningning Bai,[1]† Liu Wang,[1,2]† Qi Wang,[1] Jue Deng,[1,2] Yan Wang,[1] Peng Lu,[1] Jun Huang,[1] Gang Li,[1] Yuan Zhang,[1] Junlong Yang,[1] Kewei Xie[1], Xuanhe Zhao[2], Chuan Fei Guo[1,3]*

[1]Department of Materials Science and Engineering and Centers for Mechanical Engineering Research and Education at MIT and SUSTech, Southern University of Science and Technology, Shenzhen 518055, China

[2]Department of Mechanical Engineering, Massachusetts Institute of Technology, Cambridge, MA 02139, USA.

[3]Shenzhen Engineering Research Center for Novel Electronic Information Materials and Devices, Southern University of Science and Technology, Shenzhen 518055, China

† Ningning Bai and Liu Wang contributed equally to this work.

*** Corresponding author. Email:** guocf@sustc.edu.cn

**Supplementary Figures**

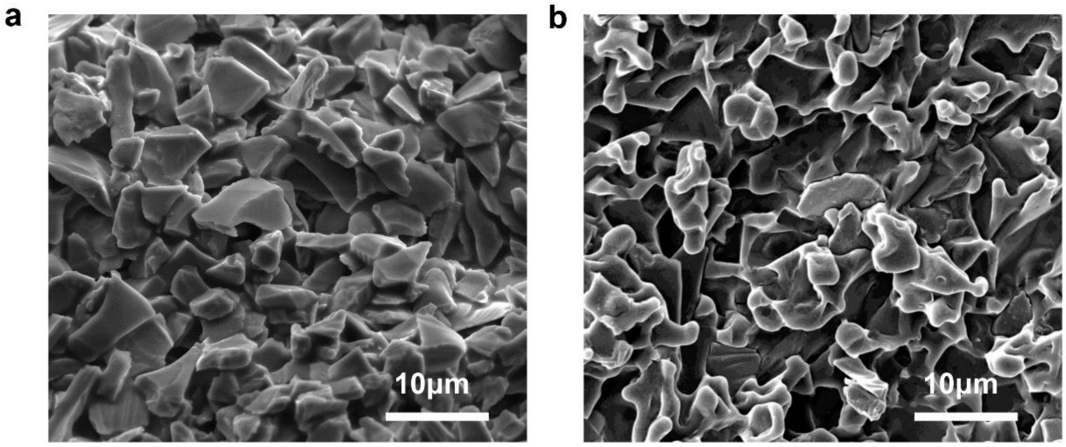


**Supplementary Figure 1. SEM images. (a)** Sandpaper template and **(b)** GIA film.

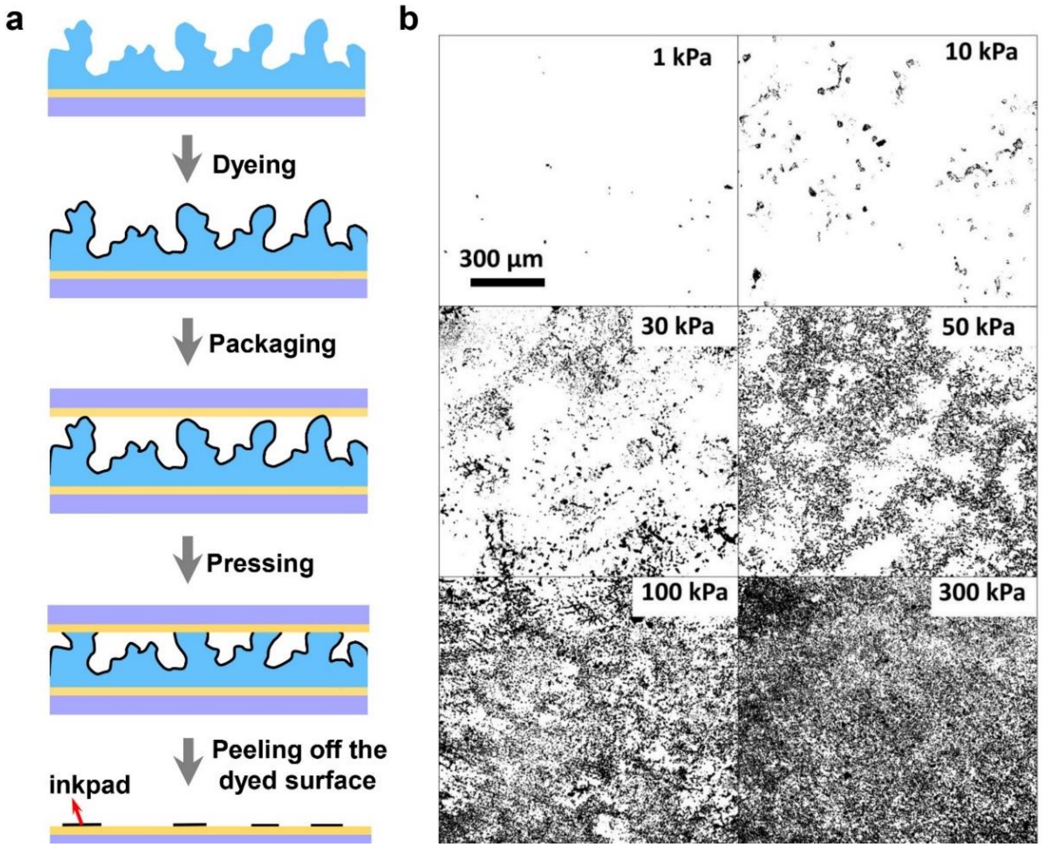


**Supplementary Figure 2. Visualization of contact area between the PVA/$H_3PO_4$ GIA film and electrode under different pressures. (a)** Diagram of dyeing of electrode upon loading. **(b)** Change of contact area between the PVA/$H_3PO_4$ GIA film and the electrode under different pressures.

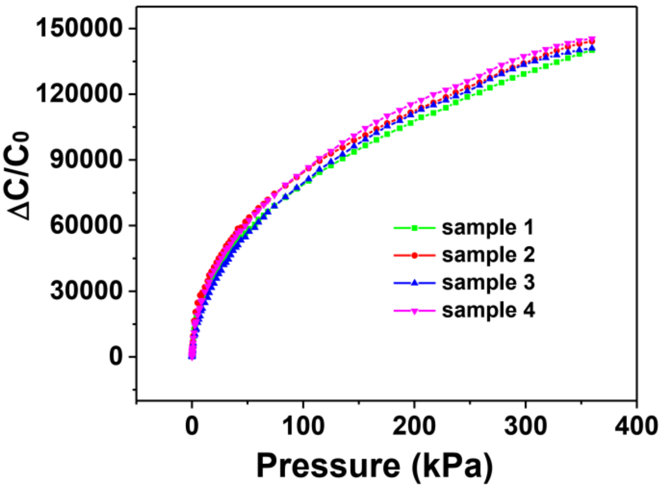


**Supplementary Figure 3. Reproducibility test for capacitance change with the pressure of the GIA-based iontronic pressure sensors in different batches.**

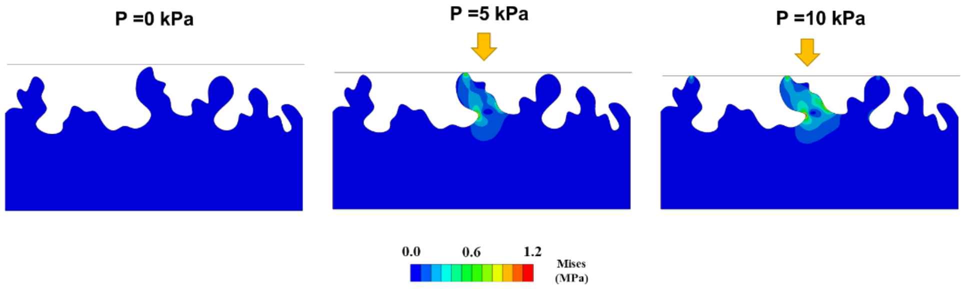


**Supplementary Figure 4. Screen snapshots of finite element simulations at *P*=0, 5, 10 kPa.**

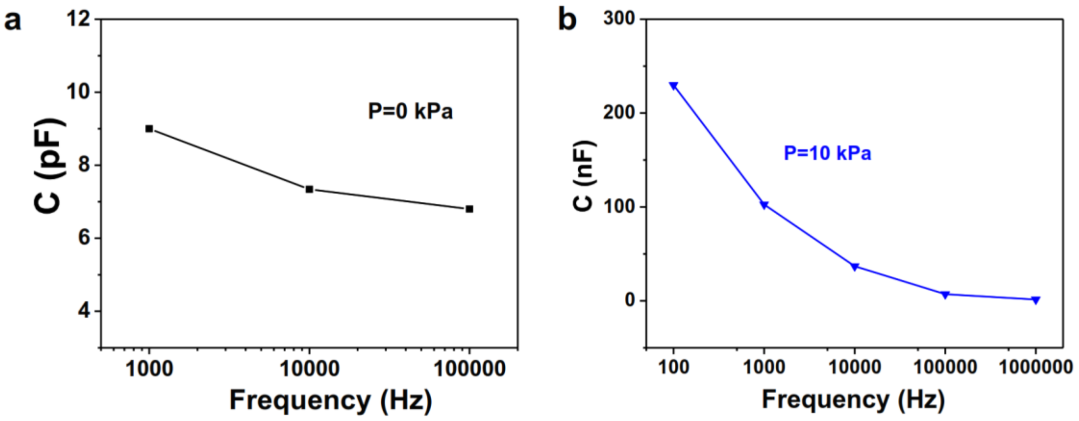


**Supplementary Figure 5. Measured capacitance as a function of frequency**. **(a)** Capacitance–frequency relation of GIA-based iontronic sensor before loading (P = 0 kPa). **(b)** Capacitance–frequency relation of GIA-based iontronic sensor under loading of 10 kPa.

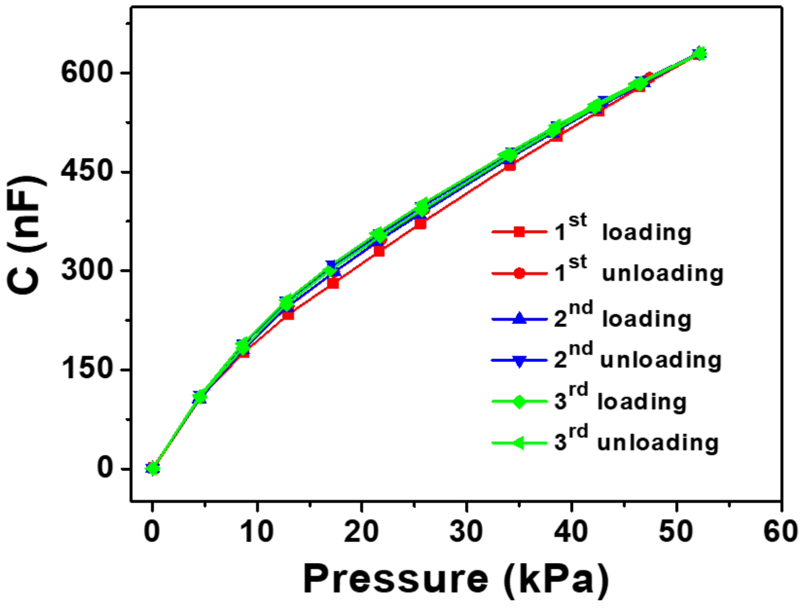


**Supplementary Figure 6. Hysteresis of the iontronic pressure sensor under three loading (50 kPa)/unloading cycles.**

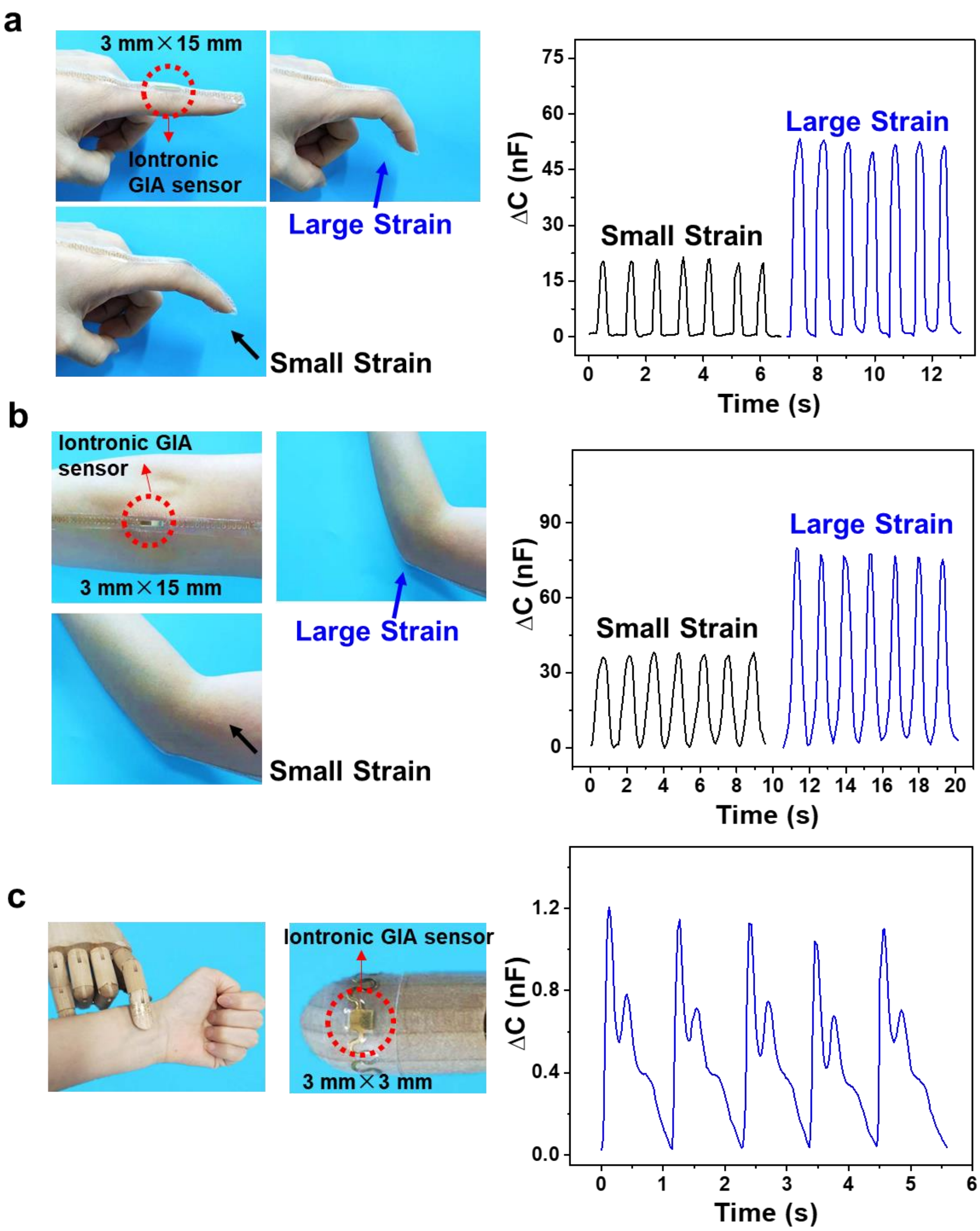


**Supplementary Figure 7. Performance of GIA-based iontronic pressure sensor.** Real-time monitoring of capacitance variations caused by repeated **(a)** forefinger bending and **(b)** elbow bending under different bending amplitude. **(c)** Pulse wave signals of the radial artery at the test frequency of 0.1 MHz.

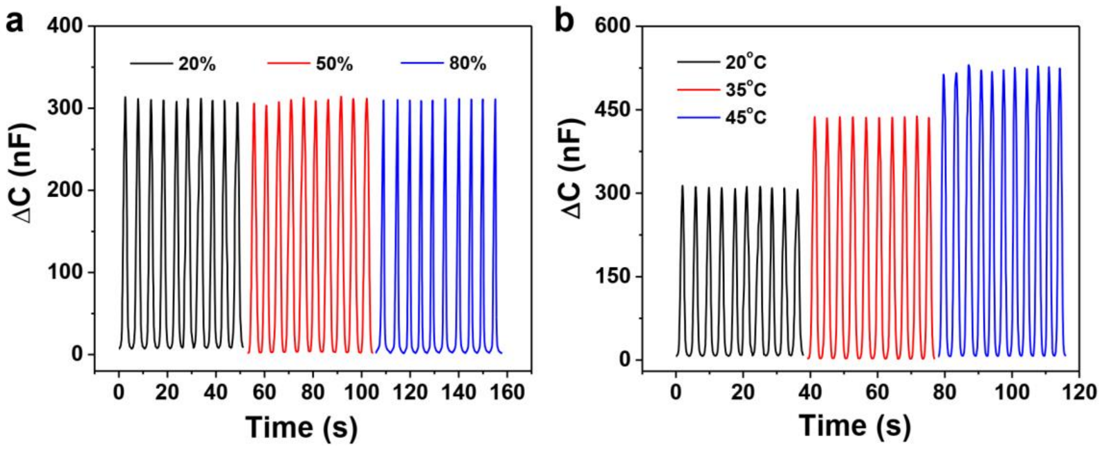


**Supplementary Figure 8. Sensing performance under different conditions. (a)** Relative humidity; **(b)** Temperatures.

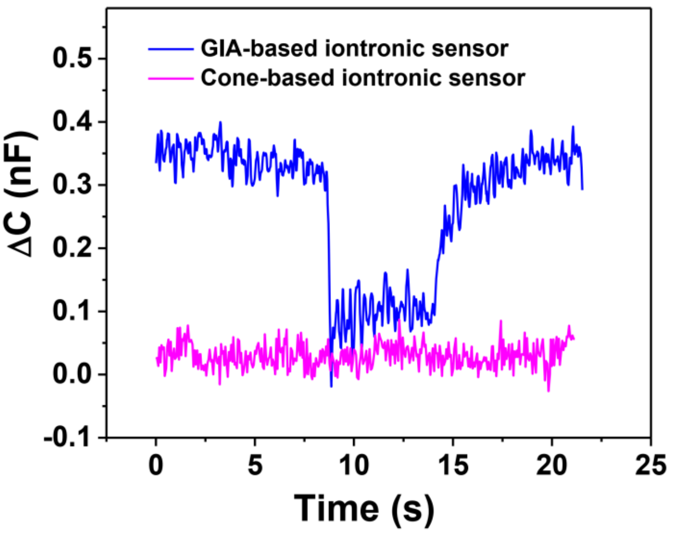


**Supplementary Figure 9. Responses by unloading from/loading on a bag of paper towel (1.7 kg) to a car trunk using iontronic sensors applying GIA and with microcones, respectively.**

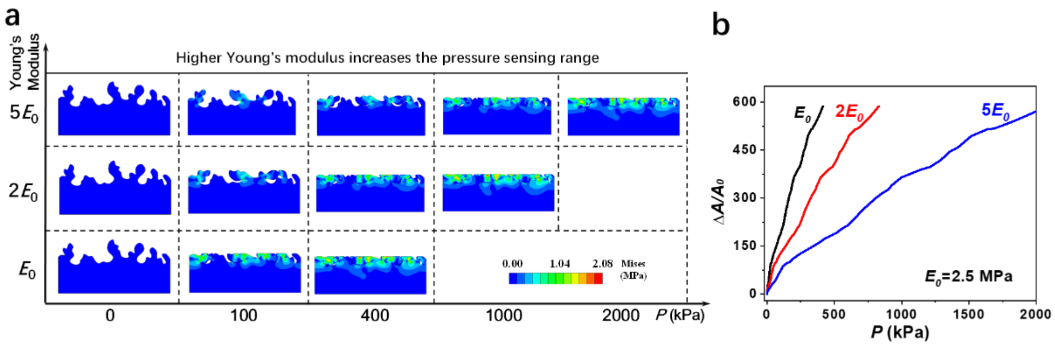


**Supplementary Figure 10. Higher Young's modulus increases the pressure sensing range. (a)** Finite element analyses of GIAs with different material moduli of $E_0, 2E_0$ and $5E_0$, where $E_0$ =2.5 MPa. When Young's modulus gets 5 times higher, the pressure sensing range can reach up to 2000 kPa. **(b)** Corresponding simulation results for normalized contact area as a function of applied pressure.

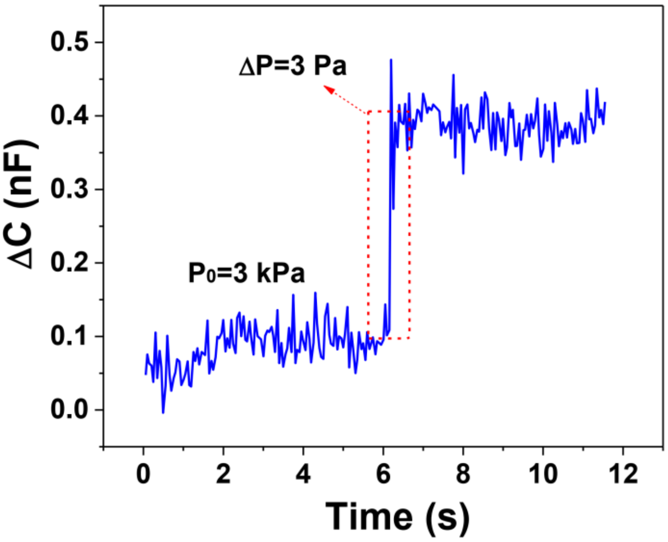


**Supplementary Figure 11. Resolving a tiny pressure change of 3 Pa under a basic pressure of 3 kPa.**

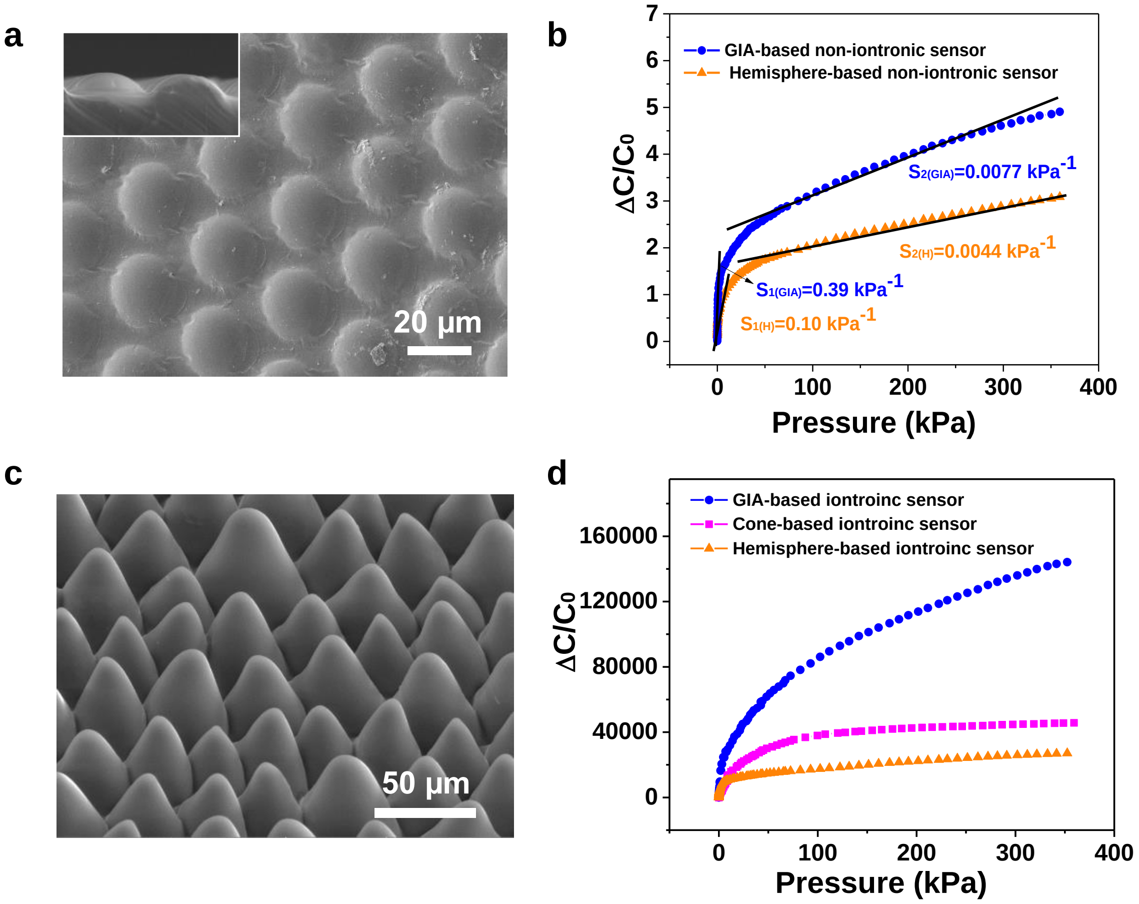


**Supplementary Figure 12. Performance comparison among sensors applying the GIA, microstructured hemispheres and cones. (a)** SEM image of the microstructured hemispheres. **(b)** Comparison of the change in capacitance with the pressure between GIA-based and hemisphere-based non-iontronic pressure sensors. **(c)** SEM image of the microstructured cones. **(d)** Comparison of the change in capacitance with increasing pressure among GIA-based, hemisphere-based, and cone-based iontronic pressure sensors.

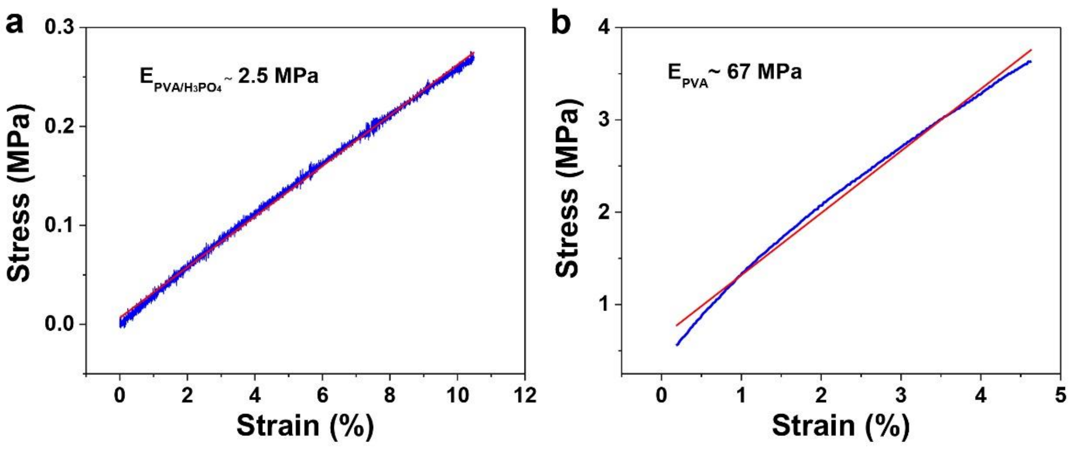


**Supplementary Figure 13. Experimental stress-strain curves**. **(a)** PVA/$H_3PO_4$ film and **(b)** PVA film. Blue lines are experimental data and red lines are fitted.

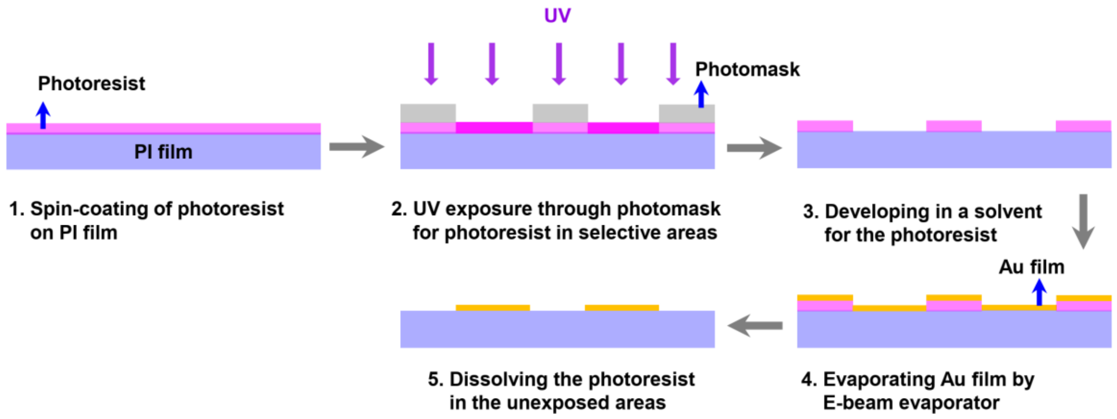


**Supplementary Figure 14. Fabrication process of micro-electrodes.**

## Supplementary Tables

**Supplementary Table 1.** Capacitance density of GIA-based iontronic sensors with different device areas.

| Device area ($\mu m^2$) | Capacitance density ($pF/\mu m^2$) |
|---|---|
| 50 × 50 | 0.417 |
| 100 × 100 | 0.411 |
| 200 × 200 | 0.412 |
