# Supplementary material for: Graded intrafillable architecture-based iontronic pressure sensor with ultra-broad-range high sensitivity
Source: Nat Commun. 2020 Jan 10;11:209. doi: 10.1038/s41467-019-14054-9 (PMC6954251; doi:10.1038/s41467-019-14054-9)
Supplement: Supplementary file 3 — Description of Additional Supplementary Files [file 41467_2019_14054_MOESM3_ESM.pdf]

Description of Additional Supplementary Files

File Name: Supplementary Movie 1
Description: Finite element analysis of a hemisphere compressed against a frictionless rigid plate.

File Name: Supplementary Movie 2
Description: Finite element analysis of a titled pillar compressed against a frictionless rigid plate.

File Name: Supplementary Movie 3
Description: Finite element analysis of an intrafillable pillar without gradient compressed against a frictionless rigid plate.

File Name: Supplementary Movie 4
Description: Finite element analysis of a GIA compressed against a frictionless rigid plate.
